# Progress in (Photo)electrochemical Biosensors for the Detection of Amyloid-Beta Oligomer

**DOI:** 10.3390/bios16060349

**Published:** 2026-06-22

**Authors:** Yaliang Huang, Ning Wang, Xinyao Yi, Ning Xia

**Affiliations:** 1 School of Pharmacy, Hunan University of Chinese Medicine, Changsha 410208, China; yalianghuang@hnucm.edu.cn; 2 College of Chemistry and Chemical Engineering, Anyang Normal University, Anyang 455000, China; 241006030@stu.aynu.edu.cn; 3 College of Chemistry and Chemical Engineering, Central South University, Changsha 410083, China; yixinyao@csu.edu.cn

**Keywords:** amyloid-beta oligomer, recognition element, electrochemical biosensor, electrochemiluminescence biosensor, photoelectrochemical biosensor

## Abstract

Alzheimer’s disease (AD) has become a neurodegenerative disease with an increasing incidence rate and a large economic and social burden worldwide. Amyloid-beta oligomer (AβO) has been confirmed as a key neurotoxic species and a core diagnostic biomarker in AD. Traditional methods for AβO detection have drawbacks, such as cumbersome operation, high cost, and dependence on sophisticated instruments, hindering their transformation into fast and real-time detection techniques. (Photo)electrochemical biosensors have attracted much attention due to their inherent advantages, such as high sensitivity, low cost, portability, and ease of miniaturization. This review systematically summarizes the latest progress of (photo)electrochemical biosensors for AβO detection, mainly based on two sensing modes: direct detection and sandwich-type detection. We comprehensively elaborated on the sensing performances and recognition elements, such as antibodies, aptamers, peptides, and molecularly imprinted polymers. The integration of functional nanomaterials and signal amplification strategies was emphasized to improve the sensitivity, selectivity, and stability of biosensors. In addition, we discussed the existing challenges and looked forward to the future development direction for the early diagnosis of AD. This article aims to provide a systematic reference for the rational design and practical application of advanced biosensors in biomarker detection and AD-related precision medicine.

## 1. Introduction

Alzheimer’s disease (AD) was first described by Alois Alzheimer in 1907 as a severe neurodegenerative disease [1]. Its symptoms include memory impairment, aphasia, and impairment of visual–spatial abilities. These lesions lead to progressive cognitive impairment and memory loss, thereby affecting patients’ daily lives and further causing difficulties in learning, spatial recognition, and cognitive performance [2]. The disease accounts for over 78% of cases of dementia, affecting nearly 55 million people worldwide. It is expected that by 2050, the number of dementia patients will increase to 132 million and become the second leading cause of death after cancer by 2040 [3]. By 2030, the estimated cost of dementia worldwide is expected to grow exponentially, reaching $2 trillion [4]. Therefore, it is meaningful to develop efficient analytical methods for monitoring the disease.

Pathologically, AD is characterized by extracellular amyloid-β (Aβ) plaques, intracellular neurofibrillary tangles composed of hyperphosphorylated tau protein, synaptic loss, and neuronal death. The amyloid cascade hypothesis posits that the aggregation and deposition of Aβ peptides are the primary causes of neuronal death and pathogenesis of AD [5]. Aβ peptides are produced from the sequential cleavage of amyloid precursor protein (APP) by β-secretase and γ-secretase in the amyloidogenic pathway [6]. They mainly consist of 39–43 residues with a highly hydrophilic N-terminal domain and a C-terminal hydrophobic domain. Among them, the most common and important peptides are Aβ1–40, or Aβ40, and Aβ1–42, or Aβ42, composed of 40 and 42 amino acid residues, respectively. In the biological fluids of AD patients, the actual production of Aβ1–40 is almost 10 times higher than that of Aβ1–42. However, Aβ1–42 is more prone to aggregation and shows stronger neurotoxicity in vivo than Aβ1–40, probably due to the two additional hydrophobic C-terminal amino acid residues [7,8]. Aβ peptides can aggregate into smaller oligomers (dimers, trimers, and tetramers), medium-range oligomers (hexamers, nonamers, and dodecamers), and higher-order assemblies, such as fibrils and fibers [9]. It has been suggested that neurotoxicity is not necessarily associated with the loading of fibrils [10], and small diffusible AβO as an intermediate product during the formation of fibrillar material is highly likely to be the main culprit of neuronal damage, toxicity, and other harmful effects [11]. One of the possible mechanisms for AβO-induced toxicity is its incorporation into the lipid bilayer of neuronal cells to disrupt membrane homeostasis, leading to abnormal influx of Ca^2+^ ions and excessive membrane depolarization [12]. The increased intracellular Ca^2+^ ions will accelerate the generation of reactive oxygen species, enhance neurotransmitter excitotoxicity, and promote neuronal death. In addition, AβO is believed to trigger the phosphorylation of microtubules, thereby hindering signal transmission in neurons and ultimately leading to neuronal damage. AβO has been found in the biofluids of AD patients, and its content is more correlated with disease severity than that of plaques [13,14]. Therefore, AβO can be used as an ideal early diagnostic biomarker and therapeutic target for AD.

Techniques for the detection of soluble AβO include fluorescence [15,16], enzyme-linked immunosorbent assay [17], surface plasmon resonance [18,19], surface enhanced Raman spectroscopy [20], colorimetry [21], field effect transistor [22], and mass spectrometry [23,24]. These methods are often time-consuming, expensive, and highly complex, and/or require sophisticated instruments, making them unsuitable for point-of-care or real-time monitoring. To overcome these issues, researchers have been exploring other methods to enhance detection sensitivity, selectivity, and simplicity. Electrochemical biosensors typically focus on the interface systems formed by metal electrodes and electrolyte solutions. By monitoring the progress of redox reactions or the changes in impedance and conductivity, qualitative detection and quantitative analysis of biomarkers can be achieved. Compared with optical methods, electrochemical biosensors are simple, low-cost, easily miniaturized, and compatible with portable or wearable devices. They are unaffected by sample turbidity or color, and can provide rapid, real-time readouts. Moreover, the electrical signal can be readily digitized and integrated with wireless communication, facilitating personalized healthcare and home-based monitoring. Recently, different electrochemical techniques have been widely used for determining AβO due to their low cost and ease of portability. For example, voltammetry or amperometry with a voltage as the input is usually used to measure the resulting current that arises from the electrochemical oxidation or reduction of redox-active molecules near the electrode surface. These techniques are further classified by specific potential-application methods, including linear sweep voltammetry, cyclic voltammetry, differential pulse voltammetry, square wave voltammetry, square wave stripping voltammetry, chronoamperometry, and chronocoulometry. In addition, electrochemical impedance spectroscopy (EIS), involving a small amplitude sine wave alternating current signal to the system, can measure the changes in impedance amplitude and phase angle with frequency and construct Nyquist and Bode plots to analyze the process of charge transfer and diffusion [25]. By using an equivalent circuit model, parameters such as solution resistance and charge transfer resistance can be obtained after the capture of AβO. Electrochemiluminescence (ECL) is the perfect combination of chemiluminescence and electrochemical technology, which can generate a light signal through electrode reactions. Unlike photoluminescence, ECL requires no external excitation light source, avoiding issues such as light scattering and autofluorescence from biological samples. Numerous excited-state luminophores are generated in a controlled, regenerative manner, enabling signal amplification. Thus, ECL is particularly suitable for AβO detection at very low concentrations in complicated biological fluids with the advantages of low background noise and high sensitivity [26]. Photoelectrochemical (PEC) technology utilizes semiconductor materials to drive electrochemical reactions under light, which can directly convert solar energy into chemical energy. Semiconductor photoanodes absorb photons and generate electron–hole pairs, which are separated in external circuits or electrolytes to form photocurrents [27]. In addition, nanomaterials have been used as powerful candidates for developing more sensitive biosensors as efficient signal components and basic building blocks [28,29,30,31]. Their unique dimension and shape, large surface area, excellent conductivity, and size-dependent properties provide significant advantages for the detection of AβO. Through rational design and controlled synthesis, nanomaterials with distinctive characteristics are effectively utilized to optimize the performance of biosensors, such as recognition efficiency, signal transduction capability, and detection sensitivity.

**Scheme 1:**
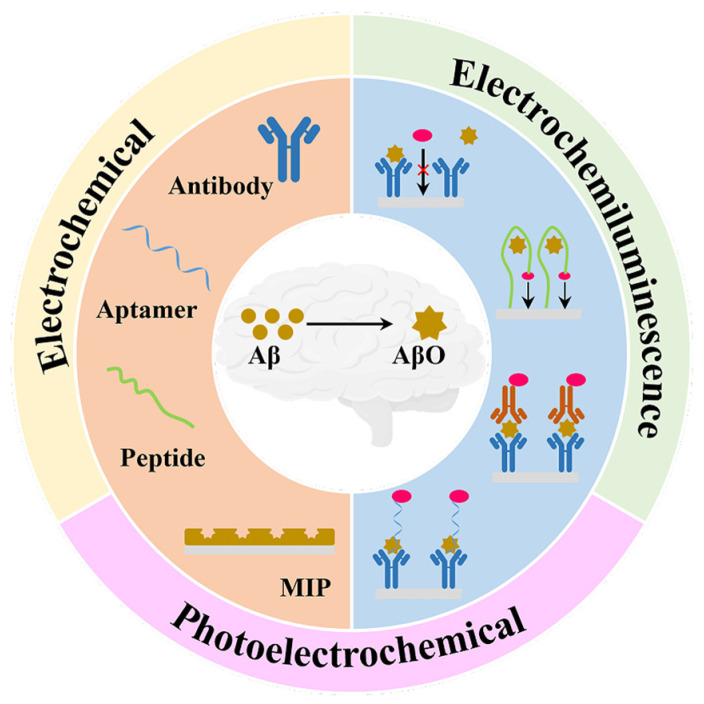
Schematic illustration of direct and sandwich-type (photo)electrochemical biosensors with antibodies, aptamers, peptides, and MIPs as recognition elements.

The analytical performance of biosensors is closely related to the recognition elements, including enzymes, antibodies, aptamers, peptides, molecularly imprinted polymers (MIPs), and others. The specific sequestration of targets by recognition elements can cause changes in physicochemical parameters such as light, heat, pH, charge, or mass, which can be sensitively detected by appropriate instruments or methods. There are some reviews on the detection of AD biomarkers in the past [32,33,34,35,36,37,38,39,40,41,42,43]. For example, Chen et al. provided an overview of interactions and biosensors of multiple AD biomarkers [44]. Sharma et al. summarized the development of electrochemical immunosensors for the detection of Aβ and Tau proteins [45]. Zamanian et al. reviewed the advances in aptamer-based biosensors for sensitive determination of AD biomarkers [46]. However, most of the existing reviews on AD biomarkers cover a broad range of analytes (including Aβ monomers, fibrils, tau, etc.) and address the detection of AβO with limited chapters. There is still a lack of specialized reviews focusing exclusively on the (photo)electrochemical detection of AβO, with systematic classification of sensing modes (direct vs. sandwich) and recognition elements (antibodies, aptamers, peptides, and MIPs). Given the importance of AβO in AD diagnosis, it is necessary to summarize the advancement of electrochemical biosensors for the determination of AβO. This article provides a comprehensive review of the (photo)electrochemical detection of AβO in terms of the types of recognition elements, focusing on two predominant sensing formats: direct detection and sandwich-type detection. We elaborate on the classification, recognition mechanism, and sensing capability of various recognition elements, including antibodies, aptamers, peptides, and MIPs (Scheme 1). Special attention is paid to the integration of functional nanomaterials and signal amplification strategies, which play a crucial role in improving the sensitivity and selectivity of electrochemical biosensors. Additionally, we address the current challenges in this field and outline future development trends. This work aims to provide a comprehensive reference for rational design and practical application of advanced electrochemical biosensors for the detection of AβO and early diagnosis of AD.

## 2. Direct Detection

Before detailing the (photo)electrochemical biosensors for AβO detection, it is worthwhile to briefly compare two major sensing formats—direct (label-free) and sandwich-type—in terms of their principles, advantages, and limitations (Scheme 1). In the direct detection mode, recognition elements (antibodies, aptamers, peptides, or MIPs) are usually immobilized on the electrode surface. The binding of AβO directly blocks the electron transfer of redox mediators from the solution to the electrode, producing measurable signals without additional labels [47]. This detection format is simple, rapid, and cost-effective, and avoids the requirement of multiple recognition elements. However, its sensitivity is often relatively moderate because no signal amplification step is involved, and the signal change solely relies on the steric hindrance or electrostatic effect caused by target binding. In contrast, sandwich-type detection methods (e.g., antibody–antigen–antibody or aptamer–target–aptamer) utilize a pair of recognition elements: a capture probe on the electrode and a detection probe conjugated with signal reporters (enzymes, electroactive molecules, or nanomaterials) [48]. The formation of the sandwich structure brings the reporters to the electrode surface, generating a significantly amplified signal. This mode offers superior sensitivity, specificity, and the possibility of multi-signal amplification (e.g., via nanoparticles, DNA nanotechnology, or enzymes). Nevertheless, it requires two well-matched recognition elements that bind to different epitopes of AβO, which may not always be available. The fabrication process is more complex, time-consuming, and costly, and may suffer from higher batch-to-batch variability. Thus, the choice between direct and sandwich-type methods depends on the specific application. Direct detection is suitable for rapid screening or when simplicity and low cost are prioritized, while sandwich assays are preferred for ultrasensitive quantification of trace AβO in complex biological samples. Comfortingly, nanomaterials play multiple roles in direct detection for improved detection performance (Table 1). For example, porous metal nanoparticles, MXene, metal–organic frameworks (MOFs), and covalent organic frameworks (COFs) can increase the surface area of sensor electrodes for probe loading. Gold nanoparticles (AuNPs) and carbon nanomaterials can enhance signal intensity by accelerating the electron transfer of redox mediators. In addition, zwitterionic peptides and polymers can provide antifouling properties to reduce nonspecific adsorption. More interestingly, nanomaterials can serve as ECL or PEC emitters generating strong, low-background signals. Recognition elements with high selectivity and specificity toward various analytes are of great importance in the development of high-performance biosensors [49,50,51]. They can specifically recognize and capture targets on the electrode interface to produce a detectable signal and reflect the change in target concentration. According to the types of recognition elements used for AβO detection, this section is discussed from four classes: antibodies, aptamers, peptides, and MIPs (Table 1).

### 2.1. Antibodies

Antigens are invaders such as viruses and bacteria, while antibodies are proteins specifically produced by the body to precisely combat invaders. Antibodies and antigens are like a perfect match of “lock and key”, and their specific binding is the cornerstone of immunoassays. Usually, antibodies can be immobilized on the electrode surface through various strategies, including physical adsorption, covalent cross-linking, and bioaffinity. The classic electrochemical immunosensors can be divided into two categories: direct immune sensors and “antibody–antigen–antibody” sandwich immune sensors [29]. In the direct detection mode, when the antigen is captured by the antibody on the electrode, the electron transfer of the redox probe from the electrolyte to the electrode surface is hindered, leading to a change in the electrochemical signal. The amount of antigen can be quantitatively determined by different electrochemical techniques or methods. The application of direct immunoassays for AβO detection is discussed in this section.

**Figure 1:**
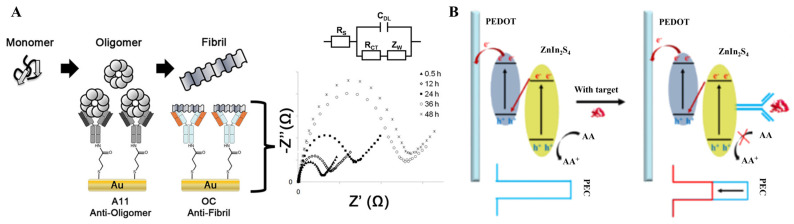
(**A**) Detection principle for monitoring Aβ1-42 fibrils and toxic oligomers using conformation-specific antibodies in conjunction with EIS. Reprinted with permission from ref. [55]. Copyright 2014 American Chemical Society. (**B**) Schematic diagram of charge transfer mechanism and signal change at the PEC sensor electrode interface. Reprinted with permission from ref. [56]. Copyright: 2025, Elsevier.

EIS has been widely used for the characterization of electrode modification and quantitation of analytes [52]. Impedimetric detection can be performed in aqueous solutions with additional redox species, such as [Fe(CN)_6_]^3−/4−^ and [Ru(NH_3_)_6_]^3+/2+^. In typical EIS methods for AβO detection, the obtained impedance spectra are fitted using an appropriate equivalent circuit model to extract meaningful interfacial parameters. The most commonly used model is the Randles circuit, which consists of solution resistance (*R*_s_), charge transfer resistance (*R*_ct_), constant phase element or double-layer capacitance (*C*_dl_) representing the electrode/electrolyte interface, and sometimes Warburg impedance (*Z*_w_) accounting for diffusion-limited processes. Among these, *R*_ct_ reflects the electron transfer kinetics of redox probes at the electrode surface. When AβO binds to the recognition element (antibody, aptamer, etc.), the formation of an insulating biomolecular layer will hinder the electron transfer, leading to an increase in *R*_ct_. Therefore, *R*_ct_ is typically used as the quantitative signal for the label-free direct detection of AβO [53,54]. For example, Veloso et al. reported an electrochemical immunosensor for the label-free evaluation of the aggregation process of Aβ1–42 into oligomers and fibrils, as well as for screening the efficacy of aggregation modulators (Figure 1A) [55]. In this study, conformation-specific antibodies (anti-oligomer A11 antibody and anti-fibril OC antibody) were covalently immobilized on the surface of a gold compact disk electrode via a 3,3′-dithiobis(sulfosuccinimidyl) propionate (DTSSP) linker for the recognition of AβO and Aβ fibril. In the presence of [Fe(CN)_6_]^3−/4−^ as the redox probe, oligomers or fibrils of Aβ1–42 specifically bound to the corresponding antibodies to form antigen–antibody complexes, leading to the increase in *R*_ct_ on the electrode surface. The time-dependent formation process of oligomers and fibrils was quantified by EIS, and the efficacy of two Aβ aggregation modulators was successfully evaluated. The method could be used to determine AβO and Aβ fibril in a direct detection mode. Zwitterionic peptides with excellent properties of hydrophilicity and electrical neutrality have been widely applied in the modification of transducer interfaces to minimize non-specific adsorption or biofouling. Huet al. developed an anti-fouling PEC immunosensor based on a Z-scheme heterojunction and zwitterionic peptide for the sensitive detection of AβO (Figure 1B) [56]. In this work, poly(3,4-ethylenedioxythiophene) (PEDOT) was modified on the surface of an indium tin oxide (ITO) electrode via electropolymerization, and then ZnIn_2_S_4_ nanosheets were loaded to construct a Z-scheme PEDOT/ZnIn_2_S_4_ heterojunction. PEDOT with a suitable bandgap could efficiently separate photogenerated electron–hole pairs and enhance the photoelectric response intensity. After the modification of the antibody, a zwitterionic peptide was covalently attached to the sensor surface to minimize the non-specific adsorption. Upon light excitation, electrons were promoted from the valence band to the conduction band. Then, the photogenerated electrons in the conduction band of ZnIn_2_S_4_ recombined with the photogenerated holes in the valence band of PEDOT. The photogenerated electrons in the conduction band of PEDOT flowed to the electrode and generated the photocurrent, and the photogenerated holes retained in the valence band of ZnIn_2_S_4_ participated in the oxidation reaction of ascorbic acid (AA). When AβO was specifically captured by its antibody, the resulting immuno-complex impeded the reaction between the photogenerated hole on the electrode surface and the electron donor of AA in the aqueous solution, leading to a significant decrease in photocurrent. Therefore, the optimized PEC immunosensor exhibited a linear detection range of 0.01~5 ng/mL and a detection limit of 4.7 pg/mL. It has high selectivity for interfering substances such as Aβ monomer and phosphorylated tau protein in human serum samples, providing an efficient tool for the accurate detection of AβO in complex samples.

**Figure 2:**
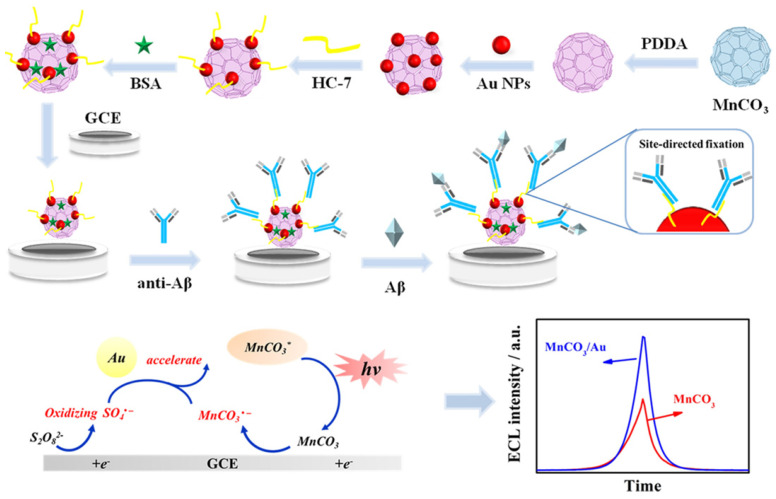
Schematic illustration of the immunosensor for AβO determination using MnCO_3_ as an ECL emitter. Reprinted with permission from ref. [57]. Copyright: 2019, American Chemical Society.

By integrating the merits of electrochemical and chemiluminescence methods, ECL biosensors show the advantages of better controllability, a wide linear detection range, and good anti-interference. They have become a research hotspot in different fields of disease diagnosis, food safety, environmental monitoring, and so on [26]. Luminophores are important elements in typical ECL biosensors, and many molecules and nanomaterials have been developed and used as ECL emitters, such as luminol, Ru(bpy)_3_^2+^, cyclometalated iridium (III) complexes, semiconductor quantum dots, and carbon dots. Jia et al. constructed a highly efficient ECL immunosensor for AβO detection using MnCO_3_ nanospheres as the emitters and a heptapeptide of HWRGWVC for site-directed immobilization of antibody (Figure 2) [57]. In this study, MnCO_3_ nanospheres prepared via a homogeneous precipitation method showed high ECL efficiency, low toxicity, and excellent biocompatibility. After functionalization with PDDA, the MnCO_3_/PDDA complex electrostatically combined with AuNPs to form the MnCO_3_/PDDA/Au composite. HWRGWVC was immobilized on the MnCO_3_/PDDA/Au surface through the Au-S bond between the cysteine residue and gold, which could effectively capture the antibody for a better maintained activity and an improved detection sensitivity. In the absence of AβO, this platform generated a strong ECL signal through the K_2_S_2_O_8_-MnCO_3_ system. Upon capture of AβO, the resulting antibody–antigen complex, serving as a non-conductive protein layer, hindered the electron transfer, thus causing a decrease in the ECL signal. Finally, this method with a “signal-off” mode achieved a linear detection range from 0.1 pg/mL to 10 ng/mL and a detection limit of 19.95 fg/mL.

### 2.2. Aptamers

**Figure 3:**
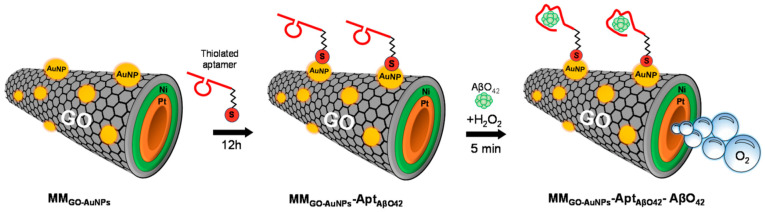
Schematic of the preparation process of M_MGO–AuNPs_–Apt_AβO42_–AβO_42_:M_MGO_/AuNPs functionalization with the specific aptamer and on-the-fly assay for AβO_42_ determination. Reprinted with permission from ref. [65]. Copyright: 2024, American Chemical Society.

Aptamer is a type of short single-stranded DNA or RNA oligonucleotide that can bind to diverse targets with high specificity and affinity [58]. Aptamers can be used as the recognition elements to replace antibodies due to their merits of simple synthesis, long shelf life, low immunogenicity, good biocompatibility, and resistance to chemical stress and high temperature. In addition, the small size of aptamers can facilitate their production and modification. Recently, aptamers have been exploited to recognize and detect Aβ species, offering novel possibilities for early diagnosis of AD [59,60]. In label-free aptasensors, the binding of AβO can cause conformational change in the aptamer and surface charge of the sensing electrode (Scheme 1), blocking the access of redox probes from the solution to the electrode surface and thus increasing the electron transfer resistance [61,62,63]. For instance, Zhang et al. developed a label-free electrochemical aptasensor for the specific determination of AβO [64]. In this work, the thiol-modified AβO-specific ssDNA aptamer was self-assembled on the surface of a gold rod electrode via the formation of an Au-S bond. Mercaptohexanol (MCH) was used to form a dense self-assembled monolayer for surface backfilling. The specific binding of AβO to the aptamer on the electrode surface hindered the charge transfer of the redox couple of [Fe(CN)_6_]^3−/4−^ to the electrode surface through steric hindrance and electrostatic repulsion, leading to a significant increase in the *R*_ct_. The quantitative analysis of AβO has been achieved by monitoring the resistance change with a wide linear detection range of 0.1~500 nM and a low detection limit of 0.03 nM. The further aggregation of oligomers into fibrils can lead to a decrease in AβO levels. By tracking the dynamic changes in AβO during the aggregation process of Aβ, the aptasensor can be used to monitor the modalities of Aβ aggregates. In addition, Gallo-Orive et al. reported a label-free electrochemical sensing platform for sensitive assays of AβO using AuNPs-based catalytic micromotors [65]. As depicted in Figure 3, AuNPs were in situ generated on the graphene oxide outer layer of tubular micromotors. Besides the covalent immobilization of AβO-specific thiolated aptamer through the Au-S interaction, AuNPs also increased the propulsion speed of micromotors by ~2 times due to their excellent catalytic activity. With the addition of AβO, the aptamer/AβO complexes on the micromotor surface produced a steric hindrance on the charge transfer of redox probes to the electrode surface, thereby causing a significant increase in *R*_ct_ and a decrease in the cathodic current of square wave voltammetry. This platform only required 5 μL of undiluted clinical samples and 5 min of detection time to realize the determination of AβO. The method showed a linear range of 0.5~500 pg/mL and a detection limit of 0.10 pg/mL. The EIS technique can be used to measure the slight change in *R*_ct_ or double-layer capacitance induced by the physical blocking of redox probes through the bound AβO-recognition element complexes. Despite the simple operation process, the sensitivity of EIS is generally moderate without signal amplification. Liu et al. designed an electrochemical aptasensor for the detection of AβO with carbon fiber paper/AuPt (CFP/AuPt) composites [66]. In this study, AuPt alloy nanoparticles were modified on a superhydrophobic carbon fiber paper by electrodeposition. The modification of AuPt increased the electroactive area and enhanced the electron transfer rate, while the hydrophobic surface improved the anti-nonspecific adsorption performance. The thiol-modified AβO-specific aptamer was self-assembled on the CFP/AuPt electrode surface. AβO specifically bound to the aptamer hindered the electron communication between ferrocene (Fc) in the solution and the electrode surface. The change in the oxidation peak current of Fc was detected by differential pulse voltammetry. Under the synergistic effect of carbon fiber paper and AuPt alloy nanoparticles, the label-free platform showed a linear range of 0.5~10,000 pg/mL and a detection limit of 0.16 pg/mL.

**Figure 4:**
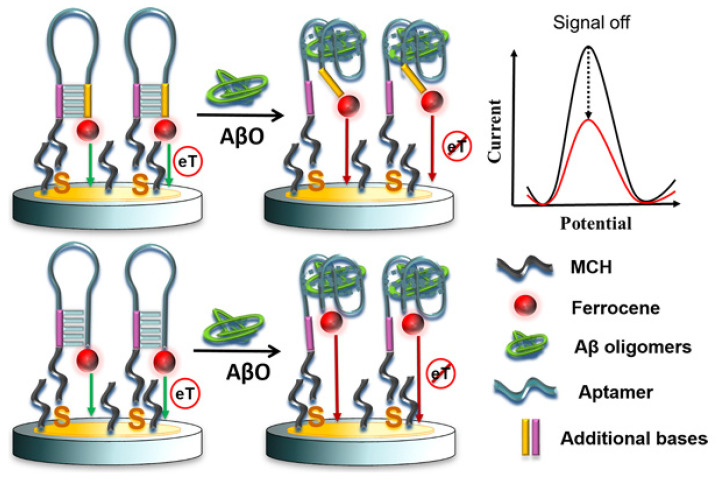
Illustrations of the mechanism for aptasensor detection of AβO based on a stem-loop aptamer. Reprinted with permission from ref. [71]. Copyright: 2019, American Chemical Society.

Redox marker can be covalently conjugated to one end of the aptamer, and the other end can be coupled with a reactive group for surface immobilization. Capture of AβO by an aptamer can lead to a change in the conformational structure of the aptamer and the distance between the redox marker and the electrode surface, inducing a change in the electrochemical signal [67,68,69,70]. Based on this principle, Zhang et al. developed a molecular beacon-based amperometric aptasensor for sensitive detection of AβO [71]. As shown in Figure 4, in the absence of AβO, the stem-loop structure was in a “closed” state and Fc was close to the electrode surface, enabling efficient electron transfer and high redox current. When the molecular beacon specifically bound to AβO, the stem-loop structure was disrupted and converted to an “open” state. The distance between the redox marker and the electrode surface increased, thus hindering the charge transfer and leading to a significant decrease in the current. Several parameters, such as stem length, oligonucleotide length, Fc-modified terminal position, and spacer, were optimized to achieve the best detection performance. Quantitative analysis of AβO was achieved by detecting the current change via alternating current voltammetry (ACV). The detection performance can be flexibly adjusted by modifying the ACV frequency. Additionally, the biosensor exhibited high selectivity for AβO with weak responses to Aβ monomers and fibrils, and good detection accuracy in artificial cerebrospinal fluids and human serum.

**Figure 5:**
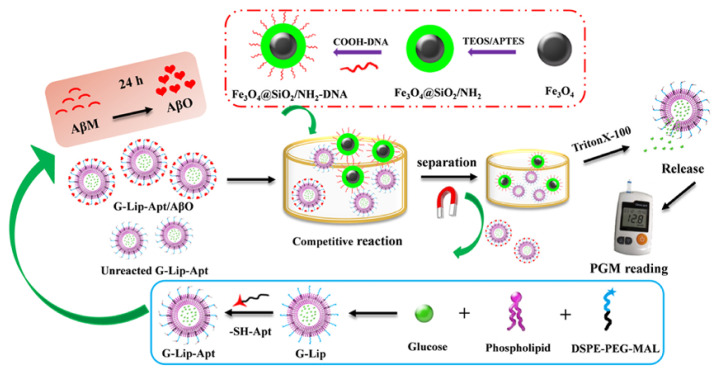
Illustrations on the preparation of aptamer grafted liposome with glucose encapsulation (G-Lip-Apt), single-strand DNA attached magnetic Fe_3_O_4_@SiO_2_/NH_2_-DNA nanocomposites, and POC sensing principle of AβO. Reprinted with permission from ref. [72]. Copyright: 2023, Elsevier.

Magnetic separation technology has the advantages of rapid and effective separation of target complexes and reduction in matrix interference in electrochemical analysis. Signal molecules encapsulated in liposomes can achieve signal amplification and protect the stability of signal molecules. The combination of the two methods can significantly improve detection sensitivity and anti-interference ability. Hu et al. fabricated a liposome-based aptasensor for electrochemical determination of AβO integrated with competitive immune-reaction, magnetic separation, and point-of-care testing [72]. As displayed in Figure 5, a liposome with a hydrophilic aqueous core was utilized to encapsulate glucose (G-Lip-Apt) and further modified with AβO-specific aptamer on the surface. Meanwhile, amino-functionalized Fe_3_O_4_@SiO_2_ nanocomposite was synthesized and conjugated with ssDNA that was partially complementary to the aptamer (Fe_3_O_4_@SiO_2_/NH_2_-DNA). AβO in samples could specifically bind to the aptamer on G-Lip-Apt, and the unreacted G-Lip-Apt could couple with Fe_3_O_4_@SiO_2_/NH_2_-DNA through the complementary interaction, facilitating the magnetic separation and removal of G-Lip-Apt. After magnetic separation, TritonX-100 was added to lyse the remaining liposome and release the encapsulated glucose, which was detected by a personal glucose meter (PGM). The PGM detection signal was negatively correlated with the concentration of AβO. Under optimized conditions, the platform exhibited a linear detection range of 5~1000 nM for AβO with a detection limit of 2.27 nM.

**Figure 6:**
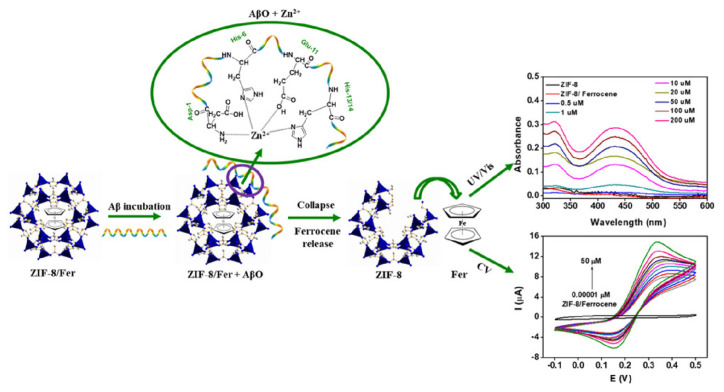
Schematic of the nanoscale ZIF-8/Fer for AβO sensing using optical and electrochemical methods. Reprinted with permission from ref. [74]. Copyright: 2019, American Chemical Society.

Nanomaterials with a hollow interior can be used as nanocontainers to load plenty of signaling molecules. The presence of a target can stimulate the sudden release of those molecules to produce a remarkably amplified signal, which is favorable in the analysis of low-abundance species with a satisfactory signal-to-noise ratio [73]. Qin et al. constructed an optical–electrochemical dual-mode biosensor for the determination of AβO based on ferrocene-encapsulated zinc zeolitic imidazolate framework (denoted as ZIF-8/Fer) (Figure 6) [74]. In this study, ferrocene with both optical and electrochemical activities was encapsulated into the pores of ZIF-8 through the self-assembly of zinc ions and 2-methylimidazole. In the presence of AβO, competitive coordination between AβO and Zn^2+^ in ZIF-8 destroyed the framework structure of ZIF-8 and triggered the release of encapsulated ferrocene molecules. The released ferrocene produced a characteristic UV absorption signal at 440 nm and obvious redox peaks, realizing the optical qualitative and electrochemical quantitative detection of AβO. Benefiting from the nanocontainer-based signal amplification strategy, this biosensor had a detection range of 10 pM~10 μM. However, ZIF-8, with relatively low stability, can also be destroyed by other biomolecules, such as ATP and amino acids, probably producing false-positive signals. To improve the stability of nanocontainers in complex samples, Ren et al. constructed an electrochemical aptasensor for AβO detection using ZIF-8-derived porous carbon as the nanocontainer and AβO-specific aptamer as the gatekeeper [75]. In this work, the specific combination of aptamer and AβO caused the opening of pores and the release of MB molecules to produce a high electrochemical signal.

**Figure 7:**
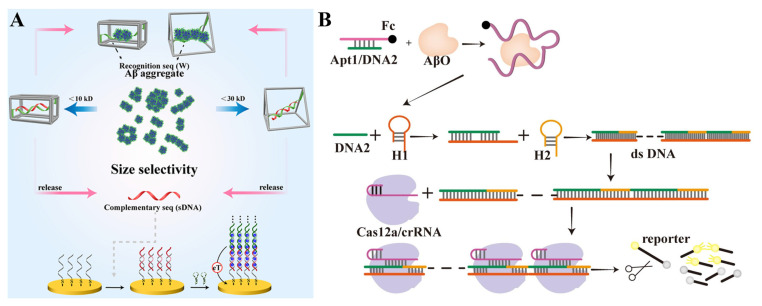
(**A**) Schematic of the DNA nanocage-assisted recognition and determination platform for LSAβO. Reprinted with permission from ref. [78]. Copyright 2024 American Chemical Society. (**B**) Schematic illustration of dual-mode detection of AβO by combining CRISPR/Cas12a gene editing technology, HCR, and nanopipettes. Reprinted with permission from ref. [81]. Copyright: 2025, Elsevier.

Taking advantage of the contemporary advancement of aptamer technology, it is available to convert the detection of AβO into the assay of DNA by coupling with diverse amplification strategies [76]. Low-mass soluble AβO (LSAβO) is more toxic, which has been considered as the potential biomarker in the clinical diagnosis of AD [77]. Chen et al. proposed a selective electrochemical method for determining LSAβO via DNA nanocage-assisted size-selective recognition and hybridization chain reaction (HCR) signal amplification strategy (Figure 7A) [78]. In this study, DNA nanocages with different sizes were investigated to achieve the best selective recognition capacity towards LSAβO. AβO-specific aptamer and its complementary sequence (sDNA) were encapsulated in the DNA nanocages. When LSAβO entered the cavity of the corresponding size nanocage, it specifically bound to the aptamer and caused the release of sDNA, triggering HCR on the electrode interface. As a result, a large number of electroactive MB probes intercalated into the base pair grooves of the formed long dsDNA, generating a strong oxidation current. This platform could be used for the selective determination of LSAβO with different sizes. To further improve amplification efficiency, several enzyme-assisted DNA amplification techniques have been designed and applied for the electrochemical detection of AβO. For instance, Liao et al. proposed a signal-on and label-free electrochemical method for the determination of AβO, in which the assay of AβO was converted into the detection of DNA based on exonuclease III-assisted DNA recycling, rolling circle amplification, and catalytic hemin/G-quadruplex for multiple-signal enhancement [79]. Cheng et al. used aptamer-modified magnetic nanoparticles to convert the presence of AβO to the generation of DNA and then initiated the nicking enzyme-powered DNA walker for the electrochemical detection of AβO [80]. Dai et al. developed a dual-signal aptasensor for fluorescent and electrochemical determination of AβO based on CRISPR/Cas12a gene editing technology, HCR, and nanopipettes (Figure 7B) [81]. In this study, dsDNA was modified on the surface of a gold-layer-deposited nanoparticle, which was formed by the hybridization of Fc-labeled aptamer (Apt1) and DNA2. Apt1 would detach from the electrode after specifically binding to AβO, resulting in a decrease in the Fc-mediated electrochemical signal. Meanwhile, the displaced DNA2 triggered the HCR to form a large number of dsDNA polymers, activating the collateral cleavage activity of CRISPR/Cas12a. A lot of Cy5-labeled reporter probes were cleaved by the activated CRISPR/Cas12a, generating an enhanced fluorescence signal. This method showed a linear range of 0.1~120 nM. More importantly, this platform could realize the qualitative detection of AβO at the single-cell level, providing an accurate and efficient method for the early diagnosis of AD.

Despite the improved sensitivity of the enzyme-assisted detection methods, the involved enzymes show relatively poor stability and may lead to a non-specific background signal. For this consideration, researchers have developed a series of enzyme-free DNA walkers for electrochemical detection of AβO by integrating with other DNA assembly techniques [82]. For example, Zhuo et al. reported an enzyme-free electrochemical aptasensor for the assay of AβO with the aid of a DNAzyme-driven bipedal DNA walker [83]. The interaction of aptamer and AβO resulted in the generation of multiple bipedal DNA walkers in the presence of corresponding molecular beacons. The formed bipedal DNA walkers could move autonomously on the hairpin DNA (MB3)-modified gold electrode under the drive of Mg^2+^-dependent DNAzyme. A large number of fragment structures were exposed on the electrode surface, and then many AgNPs were immobilized via the DNA hybridization interaction. The amount of AgNPs accumulated on the electrode could be quantitatively determined by linear sweep stripping voltammetry. The sensor had a linear range of 10 fM~0.1 nM for AβO detection with a low detection limit of 5.94 fM.

**Figure 8:**
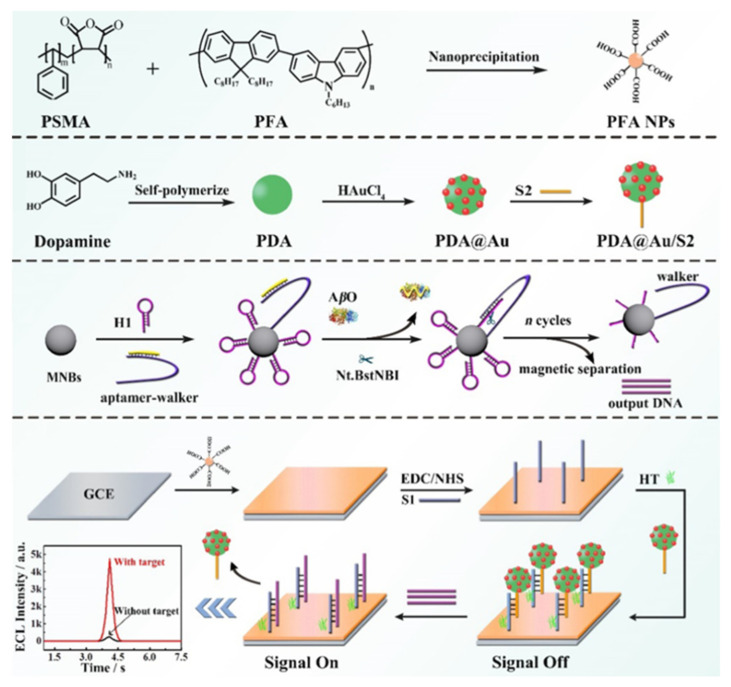
Preparation of PFA NPs, synthesis steps of PDA@Au/S2, AβO-triggered 3D DNA walker cycle amplification, and detailed assembly steps of the biosensor. Reprinted with permission from ref. [86]. Copyright: 2022, Elsevier.

By taking advantage of the competitive binding between complementary DNA and AβO toward the aptamer, it is possible to develop electrochemical bioassays [84,85]. Guo et al. developed an ECL “signal-on” aptasensor for the assay of AβO through the quenching effect of polydopamine (PDA) [86]. As displayed in Figure 8, polyfluorene nanoparticles (PFA NPs) were used as the luminophores to produce a strong ECL signal at +1.25 V with tripropylamine (TPrA) as the co-reactant. PDA was synthesized via the self-polymerization of dopamine, and AuNPs were in situ generated on the PDA surface, followed by the modification with ssDNA S2 (PDA@Au/S2). After the immobilization of PFA NPs and ssDNA S1 on the glass carbon electrode (GCE), PDA@Au/S2 was linked to PFA NPs due to the hybridization interaction. PDA as the ECL quencher efficiently quenched the luminescence of PFA NPs through dual mechanisms of resonance energy transfer and electron transfer. The detection system was performed in a “signal-off” state. The binding between AβO and the aptamer on the surface of magnetic beads triggered three-dimensional (3D) DNA walker cycle amplification with the assistance of Nt.BstNBI enzyme and releasing a large amount of output DNA. After magnetic separation, the generated output DNA competed with S2 in the S1–S2 hybrid on the electrode surface to bind S1, resulting in the release of PDA@Au/S2 from the electrode surface and the recovery of the ECL signal of PFA NPs. The detection system was switched into the “signal-on” state. The aptasensor had a linear detection range of 0.01 pM~1.00 nM with a detection limit of 8.3 fM. In addition, Jia et al. reported an ECL sensing device for visual assays of both AβO and Tau proteins based on a dual-channel closed bipolar electrode (BPE) [87]. In this study, BPE was used to connect different chambers, in which target detection and ECL generation were conducted separately to avoid cross-chemical reactions. MB-modified AβO-specific aptamers were attached to the surface of the complementary DNA-covered electrode. AβO bound to the MB-modified aptamer, and the formed AβO/aptamer complex was removed from the electrode, decreasing the current and weakening the ECL intensity of green emitter AuNCs. Conversely, the Tau protein linked the MB-modified aptamer to the electrode, leading to enhancement of current and red ECL intensity (Ru(bpy)_3_^2+^). Based on the changes in red and green signals at the anodes of the bipolar electrodes and the quantitative analysis with ImageJ 1.54r software, the device achieved a detection limit of 2.6 pM for AβO and 0.3 pM for Tau protein.

The PEC process refers to the photovoltaic–electric conversion, in which the photoactive materials can be excited by light to trigger the redox reactions between electroactive species in solution and photoexcited materials. Due to the complete separation of excitation source (light) and detectable signal (electricity), PEC biosensors exhibit unique advantages of low background signal, excellent sensitivity, ease of miniaturization, and high stability [88]. Recognition elements can be immobilized on the surface of a photoelectrode to specifically capture analytes, and the change in the photocurrent intensity can be determined. In order to amplify the signals, different strategies have been integrated with PEC techniques, such as enzyme/nanozyme catalysis, DNA assembly techniques, and in situ growth or etching strategies [89]. On account of these detection principles, PEC biosensors have been used to sensitively determine AβO in recent years [90]. Photoactive material is the key element in a high-performance PEC biosensor, and the regulation of photoactive materials can provide a sensitive PEC response for bioanalysis [27]. Zhang et al. constructed a cathodic PEC aptasensor based on a CuO/g-C_3_N_4_ p-n heterojunctioned photocathode and MoS_2_ quantum dots@copper nanowires (MoS_2_ QDs@Cu NWs) multifunctional signal amplifier for the detection of AβO [91]. First, the CuO/g-C_3_N_4_ heterojunction was prepared by in situ pyrolysis of Cu-MOF and dicyandiamide, which exhibited strong visible light-harvesting ability and high photoelectric conversion efficiency. MoS_2_QDs@Cu NWs were prepared by electrostatic self-assembly, possessing both localized surface plasmon resonance (LSPR) sensitization and peroxidase-like nanozyme activity. The sensor was immobilized on the photocathode surface through the hybridization of complementary DNA (cDNA) and MoS_2_QDs@Cu NWs-labeled aptamer. In the absence of AβO, the nanozyme catalyzed the oxidation of 4-chloro-1-naphthol (4-CN) by H_2_O_2_ to generate insoluble precipitation, hindering electron transfer and resulting in a “closed” photocurrent. When AβO specifically bound to the aptamer, dsDNA dissociated, MoS_2_ QDs@Cu NWs detached from the electrode surface, and the formation of precipitation was limited, recovering the electron transfer and turning on the photocurrent. Under optimal conditions, the biosensor showed a linear detection range of 10 fM~500 nM for AβO with a detection limit of 5.79 fM. The method showed good reliability in human serum samples, providing a new tool for the early and accurate diagnosis of AD.

**Figure 9:**
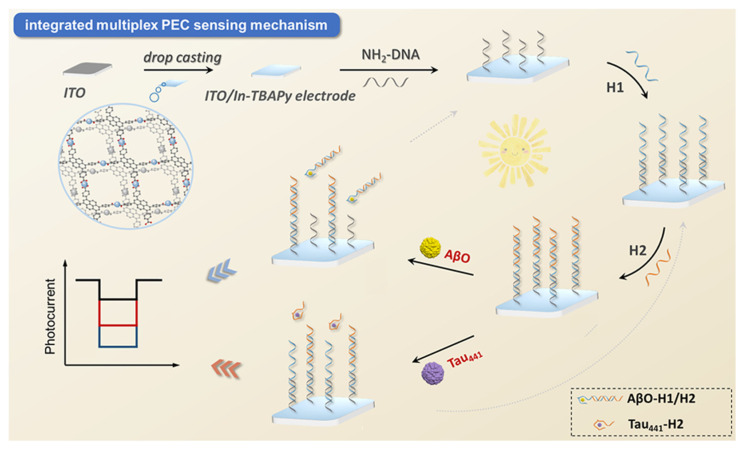
Illustration of the integrated multiplex PEC sensing mechanism for AβO and Tau 441 concatenated DNA molecular logic circuit. Reprinted with permission from ref. [92]. Copyright: 2025, American Chemical Society.

Simultaneous detection of multiple AD biomarkers is essential for AD diagnosis. To achieve the rapid recognition and simultaneous monitoring of AβO and Tau protein, Gao et al. constructed a multiplex PEC aptasensor based on concatenated DNA logic operations (Figure 9) [92]. In this study, an In-TBAPy MOF photocathode with a large π-conjugated structure, efficient visible light harvesting, and photoelectric conversion capabilities was first prepared as the sensing substrate. H1, containing an AβO-binding aptamer, and H2, containing a Tau-binding aptamer, were sequentially added to hybridize with the corresponding complementary DNA immobilized on the electrode surface, decreasing the photocurrent in the “off” state. In the presence of AβO or Tau protein, the aptamer specifically bound to the target and then detached from the electrode surface. The reduced steric hindrance enhanced the separation and transport efficiency of photogenerated carriers, resulting in a significant increase in cathodic photocurrent (“on” state). Using AβO and Tau as the inputs, the PEC biosensor constructed a dual-input logic gate, and the presence of the target could output a high current signal. The detection limits were found to be 4.47 fg/mL for AβO and 0.34 pg/mL for Tau. Nevertheless, nucleases existing in body fluids are capable of cleaving the phosphodiester bonds within nucleic acid chains, thereby disrupting the primary structure of aptamers. Metal cations have the potential to alter the conformation and functional properties of aptamers. All these constraints impede the application of nucleic acid molecules as diagnostic and therapeutic agents.

### 2.3. Peptides

**Figure 10:**
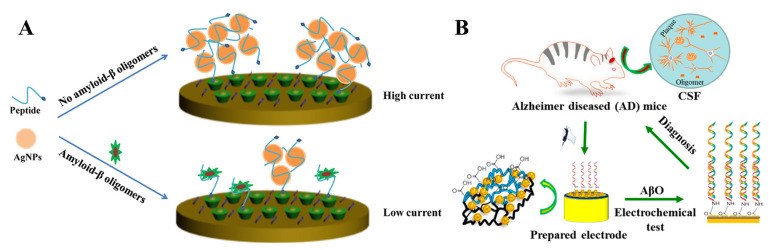
(**A**) Schematic illustration of the electrochemical method for the selective detection of AβO using AgNPs as the redox reporters and Ad-labeled PrP_95–110_ as the receptor. Reprinted with permission from ref. [102]. Copyright 2016 American Chemical Society. (**B**) Schematic illustration of the EIS method for the detection of AβO using PrP_95–110_ as the receptor. Reprinted with permission from ref. [103]. Copyright: 2019, American Chemical Society.

Peptides composed of amino acids can mimic the domains of proteins or antibodies and substitute them in protein–protein interactions or antibody–antigen immune responses. Selecting a well-matched sequence is a critical factor in developing peptide-based detection platforms. Peptide recognition elements with high affinity can be identified through natural sources, combinatorial phage display libraries, and integration of phage peptide display screening with computational modeling. Peptides exhibit unique advantages, including diverse amino acid types, flexible structures, and tunable hydrophobic/hydrophilic properties. In addition, peptides have a smaller size than antibodies, which can facilitate the entry of signal probes into the sensing surface to generate high signals [93]. Therefore, peptides have been widely utilized to develop biosensors for the accurate analysis of various biomarkers [94]. It was reported that a segment of cellular prion protein (THSQWNKPSKPKTNMK, named PrP_95–110_) can specifically bind to AβO with higher affinity than that of Aβ fibrils and monomers [95]. Since then, peptide-based electrochemical biosensors have been developed for AβO detection by several groups [96,97,98,99,100,101]. Peptides with appropriate amino acid sequences can trigger the in situ assembly of themselves and nanomaterials on the sensing interface to enhance the signals. Our group reported two electrochemical biosensors for the assays of AβO based on PrP_95–110_−triggered in situ aggregation of AuNPs or AgNPs [100,102]. As depicted in Figure 10A, adamantane (Ad)-labeled PrP_95–110_ peptide could be captured by β-cyclodextrin (β-CD)-decorated electrode via the host-guest interaction between Ad and β-CD. Meanwhile, PrP_95–110_ with positively charged amino acid residues induced the aggregation of negatively charged AgNPs on the electrode surface through electrostatic interactions. AgNPs in the formed networks generated a strong electrochemical signal through the solid-state Ag/AgCl reaction. However, binding of AβO with PrP_95–110_ hindered the aggregation of AgNPs due to the formation of AβO–PrP_95–110_ complexes, thus leading to a decreased electrochemical signal. Under optimal conditions, this biosensor showed a linear range of 0.02~100 nM and a detection limit of 8 pM. In addition, Lee’s group reported a label-free impedimetric biosensor for the specific detection of AβO by immobilizing aminated PrP_95–110_ on the surface of poly(3,4-ethylene dioxythiophene)-capped gold electrode (Figure 10B) [103]. The binding of AβO to the peptide blocked the electron transfer of redox probes to the electrode surface, resulting in an increase in R_ct_. This label-free biosensor exhibited a good linear response for AβO with a detection limit of 10 nM.

### 2.4. Other Methods

**Figure 11:**
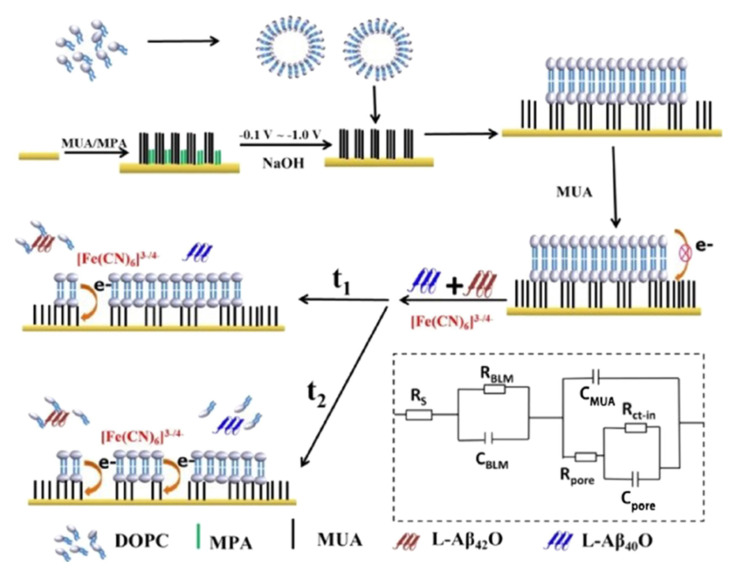
Schematic illustration for EIS detection of L-Aβ_42_O and L-Aβ_40_O. Reprinted with permission from ref. [105]. Copyright: 2021, American Chemical Society.

The same hydrophilic segment of Aβ_42_ and Aβ_40_ makes it difficult to effectively differentiate these oligomers (denoted as L-Aβ_42_O and L-Aβ_40_O). In addition, other proteins with β-sheet secondary structure may influence the selectivity of aptamer-based biosensors for AβO detection. It has been reported that both L-Aβ_42_O and L-Aβ_40_O can associate with lipid bilayers and cause leakage of model membranes, while L-Aβ_42_O displays stronger affinity and faster binding kinetics than L-Aβ_40_O in the membrane damage process [104]. Based on this fact, Duan et al. developed a specific recognition element-free electrochemical biosensor for the differentiation and detection of L-Aβ_42_O and L-Aβ_40_O according to the dynamic difference in membrane damage induced by the two oligomers (Figure 11) [105]. In this method, a mixed MUA/MPA self-assembly monolayer (SAM) was assembled on the gold electrode surface, and then MPA was removed by a selective electrochemical desorption process to form a porous MUA layer. This led to the formation of a planar bilayer lipid membrane (BLM) on the porous MUA layer-modified electrode. L-Aβ_42_O and L-Aβ_40_O caused membrane damage at different rates, which could be recorded by EIS in one chip. L-Aβ_42_O, exhibiting stronger hydrophobicity and higher affinity for BLM, could rapidly cause membrane damage and lead to a significant decrease in *R*_ct_ within 20 min. However, L-Aβ_40_O damaged the membrane at a slower rate, and the signal stabilized at 60 min. The weakly charged surface of the BLM reduced the non-specific adsorption of interfering proteins in cerebrospinal fluids. This biosensor had a linear detection range of 5~500 pM for L-Aβ_42_O with a detection limit of 3 pM.

In short, antibodies show the highest specificity but suffer from batch variation, poor stability, and high cost. Aptamers offer excellent reproducibility, stability, and moderate cost, although non-specific binding remains a problem. Peptides are cheap and stable, but their specificity is only moderate. MIPs are highly stable, cheap, and reproducible, yet their selectivity for large molecules like AβO remains a challenge. For clinical applications that require high specificity, antibodies or high-quality aptamers are preferred, and yet peptides and MIPs are more suitable for point-of-care or on-site deployment.

**Table 1:** Performance of different modified electrodes for direct detection of AβO.

Acceptor	Electrode	Linear Range	Detection Limit	Sample	Ref.
Antibody	ZnIn_2_S_4_/PEDOT/Au/ITO	10 pg/mL–5 ng/mL	4.7 pg/mL	Serum	[56]
	MnCO_3_/PDDA/Au/GCE	0.1 pg/mL–10 ng/mL	19.95 fg/mL	CSF	[57]
Aptamer	AuNPs/SPCE	10 pg/mL–100 μg/mL	2.3 pg/mL	Serum	[61]
	SAM/AuR	0.1–500 nM	0.03 nM	A-CSF	[64]
	M_MGO–AuNPs_	0.5–500 pg/mL	0.1 pg/mL	CSF	[65]
	Fc-aptamer/CFP/AuPt	0.5–10000 pg/mL	0.16 pg/mL	Serum	[66]
	3D-GMEs	1 pM–200 nM	0.3 pM	A-CSF	[67]
	GNP-STPs	1 fM–10 pM	0.5 fM	Serum	[68]
	Fc-DNA/AuE	1 fM–10 pM	0.47 fM	A-CSF	[69]
	Fc-aptamer/AuE	0.1pM–10 nM	6.5 fM	A-CSF	[71]
	PGM	5–1000 nM	2.27 nM	Serum	[72]
	DNA/GCE	10 fM–10 μM	3.4 fM	Serum	[73]
	AuE	10 pM–100 μM	10 pM	A-CSF	[74]
	DNA/AuNPs/GCE	50 fM–10nM	1.58 fM	Serum	[75]
	DNA/AuE	10–150 pM	8 pM	Serum	[78]
	Au-coated nanopipette	0.1–120 nM	2.15 pM	Serum	[81]
	DNA/AuE	10 fM–100 pM	5.94 fM	Serum	[83]
	Hairpin/IrNRs/GCE	1 pM–10 nM	0.62 pM	A-CSF	[84]
	PDA@Au/S2/GCE	0.01 pM–1 nM	8.3 fM	Serum	[86]
	Aptamer/Au-BPE	1 pM–200 nM	2.6 pM	Serum	[87]
	Thionine/MoS_2_ QDs/Cu NWs	5 fM–500 nM	2.1 fM	Blood	[90]
	CuO/g-C_3_N_4_/ITO	10 fM–500 nM	5.79 fM	Serum	[91]
	DNA/ITO	100 fg/mL–100 ng/mL	4.47 fg/mL	A-CSF	[92]
Peptide	PPy-3-COOH/AuE	0.001 fM–10 nM	1 aM	Blood	[96]
	PPyCOOH/AuE	0.1 fM–10 nM	0.1 fM	CSF	[97]
	AuS/GCE	5–200 pM	2 pM	Buffer	[98]
	GO/GNPs hydrogel	0.1 pM–10 nM	0.1 pM	Blood	[99]
	Peptide/AuE	20 pM–100 nM	8 pM	Serum	[100]
	POPA co-polymer/AuE	0.5 pM–1000 nM	0.5 pM	Buffer	[101]
	AgNPs	20 pM–100 nM	8 pM	Serum	[102]
	PTAA-AuNPs-PEDOT/AuE	0.01 fM–10 μM	0.01 fM	CSF	[103]

Abbreviations: PEDOT, poly(3,4-ethylenedioxythiophene); ITO, indium tin oxide; PDDA, polydimethyldiallylammonium chloride; GCE, glass carbon electrode; SPCE, screen-printed carbon electrode; SAM, self-assembled monolayer; AuR, gold rod electrode; M_MGO–AuNPs_, graphene oxide−gold nanoparticles; CFP, carbon fiber paper; 3D-GME, microelectrodes modified with electrodeposited 3D nanostructures; AuE, gold electrode; PGM, personal glucose meter; IrNRs, iridium(III) nanorods; PDA, polydopamine; BPE, bipolarelectrode; GNP, gold nanoparticles; STP, signal transduction probe; Fc, ferrocene; g-C_3_N_4_, 2D layered graphitic carbon nitride; PPy-3-COOH, poly(pyrrole-3-carboxylic acid); PPyCOOH, poly (pyrrole-2-carboxylic acid); AuS, gold nanostars; GO, graphene oxide; POPA, polytyramine/3-(4-hydroxyphenyl) propionic acid; PTAA, poly(thiophene-3-acetic acid); and A-CSF, artificial cerebrospinal fluid.

## 3. Sandwich Methods

(Photo)electrochemical biosensors with sandwich-like structures can be developed with well-paired receptors that can bind to different individual sites of targets, such as “antibody–antigen–antibody”, “antibody–antigen–aptamer”, “aptamer–antigen–aptamer”, etc. [106] Usually, the capture element is fixed on the electrode surface, while the recognition element is modified with signal reporters such as enzymes or nanozymes, electroactive probes, and signal molecule-loaded nanomaterials. After the formation of sandwich structures, the reporters can provide significantly amplified electrochemical signals, greatly improving detection sensitivity and selectivity (Table 2). Therefore, sandwich-type (photo)electrochemical biosensors exhibit superior advantages of high sensitivity, repeatability, and stable response, and have been widely used for the detection of different targets, including proteins, exosomes, and cells [107].

**Figure 12:**
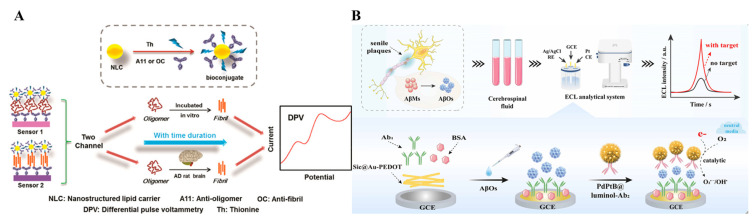
(**A**) Schematic illustration for determining Aβ oligomers and fibrils in the two-channel electrochemical system. Reprinted with permission from ref. [112]. Copyright: 2019, American Chemical Society. (**B**) Schematic illustration of the designed ECL immunosensor for AβO detection. Reprinted with permission from ref. [113]. Copyright: 2023, American Chemical Society.

Electrochemical immunosensors adopt a sandwich structure of “antibody–antigen–antibody”, in which the target is sandwiched between two functional layers, exhibiting excellent specificity, high sensitivity, and ease of automation [48]. In order to further improve detection performance, different materials have been used to label detection antibodies and amplify the signals, including electroactive molecules, enzymes, and nanomaterials [108,109,110,111]. For instance, Yu et al. constructed a dual-channel electrochemical immunosensor for simultaneous detection of AβO and Aβ fibrils using thionine-loading nanostructured lipid carriers as labels for signal amplification (Figure 12A) [112]. In this work, GCE and thionine-loading nanostructured lipid carriers were modified with anti-AβO (A11) and anti-fibril (OC), respectively, to build two sandwich immunosensors. AβO and fibrils captured by the corresponding antibodies on the electrode surface allowed for the attachment of signal probes to form sandwich structures. The platform showed a linear detection range of 0.2~40 ng/mL for both AβO and fibrils, with the detection limits of 0.01 ng/mL and 0.02 ng/mL, respectively.

In traditional ECL systems, exogenous co-reactants are always required, which may cause unstable detection results. To avoid this problem, dissolved oxygen is often used as a non-toxic endogenous co-reactant to generate reactive oxygen species (ROS) in situ under the catalysis of nanozymes as co-reaction promoters. Peng et al. proposed a novel ECL immunosensor based on PdPtB nanozyme and dissolved O_2_ for AβO detection (Figure 12B) [113]. In this work, highly conductive SiC@Au-PEDOT nanowires were used to modify the electrodes, increasing the electroactive surface area and accelerating electron transport. PdPtB mesoporous nanospheres were synthesized and subsequently modified with luminol and an antibody. In the presence of AβO, sandwich immunocomplexes were formed on the surface of SiC@Au-PEDOT. PdPtB mesoporous nanospheres with excellent oxidase-like activity could in situ catalyze the transformation of dissolved O_2_ into ROS, significantly boosting the luminol ECL signal in neutral media. The “signal-on” mode facilitated the detection of AβO down to 10 pM with a linear range of 20 pM~20 nM.

Sandwich-type immunosensors exhibit good analytical performances but still face some challenges, such as complex operation procedures, high cost, and stringent conditions for production and preservation of antibodies, as well as potential cross-reactivity with structural analogs. AβO has two or more specific recognition sites for aptamers and peptides, making it feasible to construct aptamer–analyte–aptamer sandwich biosensors. More importantly, the secondary aptamer can be connected with an appropriate DNA sequence to initiate other powerful DNA amplification processes. Such sandwich aptasensors usually require electroactive molecules or nanoparticles to increase the electrochemical response [114,115]. For example, Xu’s group employed poly(thymine)-templated copper nanoparticles and copper-based metal–organic frameworks as electroactive probes, respectively, to label the secondary aptamer for the detection of AβO [116,117].

**Figure 13:**
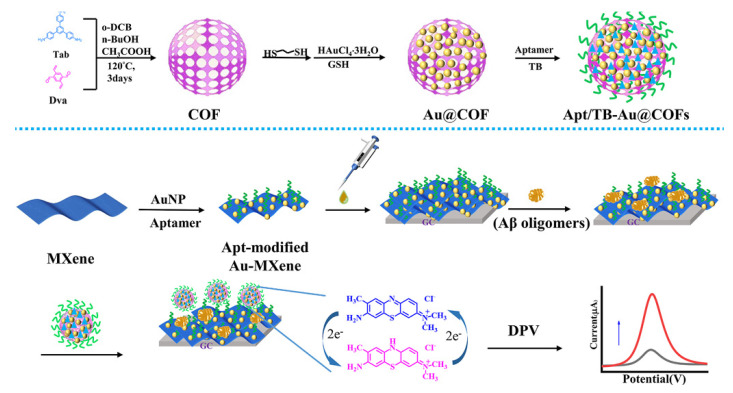
Scheme of synthesis process of the Apt/TB-Au@COFs and electrochemical determination of AβO with the Au-MXene substrate and the Apt/TB-Au@COFs. Reprinted with permission from ref. [119]. Copyright: 2024, Elsevier.

It is reported that polyadenine, consisting of multiple adenines, can bind to a gold or silver surface with high affinity. Liao et al. reported a label-free electrochemical method for AβO determination by employing HCR-triggered polyadenine to absorb silver nanoparticles (AgNPs) [118]. In this study, aptamer 1 was first immobilized on the electrode surface via the polyadenine-Au interaction. The non-specific unreacted sites were blocked with MCH. Once the addition of AβO, a sandwich structure was formed between aptamer 1, AβO, and aptamer 2. HCR was then initiated with the aptamer 2 fragment as the primer to generate long-chain double-stranded DNA suspended with an adenine-rich fragment. Polyadenine on the long-chain dsDNA could adsorb a large number of AgNPs as electroactive probes to produce a characteristic stripping current. The higher the concentration of AβO, the more polyadenine fragment generated by HCR, the more AgNPs are adsorbed, and the stronger the stripping current signal. Thanks to the dual-signal amplification of HCR and AgNPs, this label-free method exhibited a linear detection range of 1 pM~10 nM and a detection limit of 0.43 pM. In addition, Lv et al. developed an ultrasensitive electrochemical aptasensor for the detection of AβO through a dual-signal amplification strategy of MXene substrate and COF-based probe [119]. As presented in Figure 13, AuNP-decorated Ti_3_C_2_MXene composite (Au-MXene) was first prepared via an in situ reduction method. The prepared Au-MXene possessed an ultra-large specific surface area and excellent charge mobility. It was used as the electrode substrate to provide abundant binding sites for the immobilization of AβO aptamers. Meanwhile, COF was subsequently modified with AuNPs, aptamer, and toluidine blue (TB) to form the Apt/TB-Au@COF signal probe. AβO was specifically captured by the aptamer on the Au-MXene substrate and then labeled with Apt/TB-Au@COF. A large number of electroactive TB molecules in Apt/TB-Au@COF generated a high differential pulse voltammetry signal. Under optimized conditions, the platform for AβO analysis achieved a linear detection range of 0.01~180 pg/mL with a detection limit of 4.27 fg/mL. The results for the assays of clinical serum samples were highly consistent with those achieved by ELISA, providing a new, promising tool for the early clinical diagnosis of AD.

**Figure 14:**
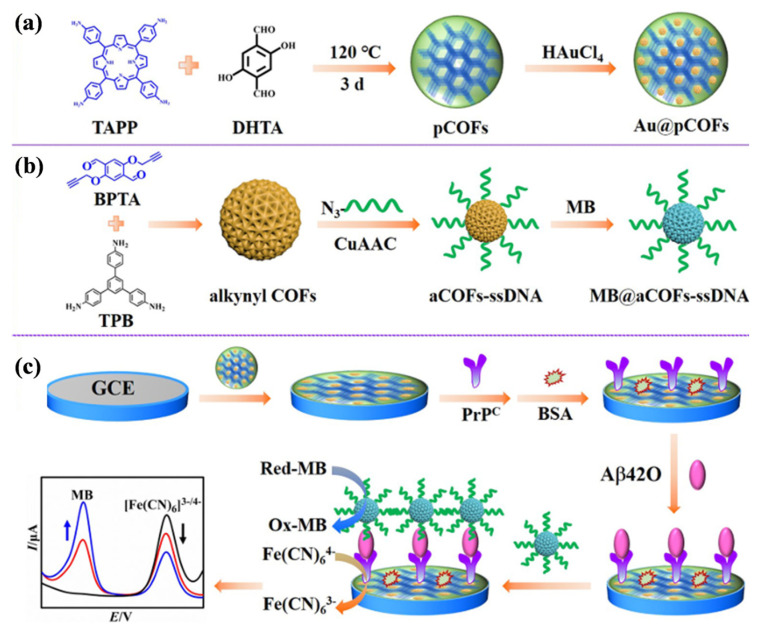
Schematic diagram of (**a**) the synthesis of Au@pCOFs; (**b**) the synthesis of MB@aCOFs-ssDNA and (**c**) a ratiometric electrochemical immunosensor for Aβ_42_O detection. Reprinted with permission from ref. [123]. Copyright: 2022, Elsevier.

In addition, the aptamer–target–peptide sandwich structure has also been developed for sensitive assays of AβO [120,121,122]. For example, Lv et al. fabricated an “aptamer–target–peptide” sandwich electrochemical biosensor for the detection of AβO using horseradish peroxidase-participated enzymatic–electrochemical redox cycling for signal amplification [120]. Liu et al. proposed a ratiometric electrochemical platform for the ultrasensitive detection of AβO through the signal amplification of COFs [123]. As shown in Figure 14, porphyrin-based COFs were modified with AuNPs and further deposited on the surface of the GCE to catalyze the redox of [Fe(CN)_6_]^3−/4−^ and immobilize the cellular prion protein (PrP^c^) via the Au–S interaction. Meanwhile, alkynyl COFs loaded with methylene blue and covalently modified with aptamers (MB@aCOFs-ssDNA) served as the signal probes. Upon specific capture of AβO by PrP^c^ on the electrode surface, the electrochemical signal from [Fe(CN)_6_]^3−/4−^ obviously decreased due to the hindrance of electron transfer. Conversely, plenty of electroactive MB molecules in the captured MB@aCOFs-ssDNA produced a strong electrochemical signal, leading to an “on–off” ratiometric mode and effectively eliminating background interference. Under optimized conditions, the platform exhibited a linear detection range of 10 pM~1 μM with a detection limit as low as 5.1 pM, and showed good stability and reproducibility for the assays of real cerebrospinal fluid samples. In addition, the sandwich structures of “antibody–target–aptamer” and “peptide–target–peptide” have been employed to design electrochemical biosensors for AβO analysis by integrating the advantages of both immune reactions and aptamer/peptides-based biosensors [124,125,126].

**Figure 15:**
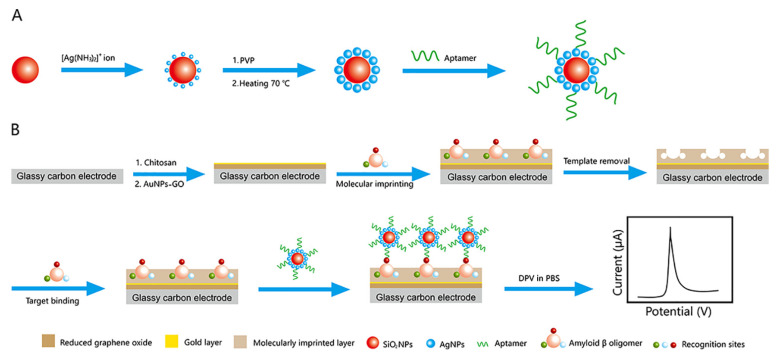
Schematic illustration of (**A**) the preparation of the SiO_2_@Ag-aptamer composite, and (**B**) a sandwich-type electrochemical biosensor for AβO detection based on MIPs and aptamers as recognition elements. Reprinted with permission from ref. [129]. Copyright: 2020, Elsevier.

MIPs are mainly formed by the polymerization of functional monomers guided by template molecules and then removing the template to create complementary cavities, which can precisely recognize and bind to specific target molecules. MIPs show the same specificity and selectivity as antibodies, which are considered “artificial antibodies”. Compared with other biorecognition elements, MIPs possess the merit of chemical stability, simple operation, excellent resistance to harsh environments, and potential applicability to all proteins [127]. The combination of MIPs with other specific but low-stability biorecognition elements (e.g., antibodies and aptamers) may endow biosensors with high specificity and sensitivity and excellent stability in a harsh detection environment [128]. You et al. developed a sandwich-type electrochemical biosensor for the highly sensitive detection of AβO using MIPs and aptamers as the recognition elements [129]. As displayed in Figure 15, an AβO-imprinted MIP film was assembled on the surface of AuNPs-GO-modified electrode to specifically capture AβO. AβO-specific aptamers were immobilized on the surface of silica@silver core–shell nanoparticles (SiO_2_@Ag NPs) as the signal probes. The amounts of AβO in samples were readily measured according to the strong electrochemical signals of electroactive AgNPs. The biosensor had a linear detection range of 5 pg/mL~10 ng/mL with a detection limit of 1.22 pg/mL.

Table 1 and Table 2 provide an overall comparison of the analytical performances of the biosensors in direct and sandwich-type detection modes, respectively. From the data compiled in Table 1 and Table 2, several trends can be observed regarding the detection limits and linear ranges of (photo)electrochemical biosensors for AβO, where the concentration of AβO was defined as that of the equivalent Aβ monomer. Sandwich-type bioassays generally provide lower detection limits compared to direct detection, attributed to the signal amplification provided by secondary probes, nanomaterials, or enzymatic reactions. Direct detection often exhibits a broader linear range than sandwich assays because sandwich sensors are more prone to signal saturation at high target concentrations due to the limited capture/detection probe stoichiometry. In brief, sandwich-type platforms consistently achieve the lowest detection limits, while direct methods offer simplicity at the cost of moderate sensitivity.

**Table 2:** Analytical performances of different electrochemical biosensors for sandwich-type detection of AβO.

Acceptor	Electrode	Signal Label	Linear Range	Detection Limit	Sample	Ref.
Antibody	Au@C_3_N_4_/GCE	CuS@CoS_2_ DSNBs@Ag NPs-Ab_2_	0.5–500 pg/mL	0.0038 pg/mL	CSF	[111]
	CNT-PIL/GCE	Th@NLC-Ab_2_	0.2–40 ng/mL	0.01 ng/mL	CSF	[112]
	SiC@Au-PEDOT NWs/GCE	PdPtB-Ab_2_	0.02–20 nM	10 pM	A-CSF	[113]
Aptamer	Aptamer/BPQDs/ITO	Aptamer-AuNRs	10 fM–100 nM	4.21 fM	Serum	[115]
	Aptamer/AuNPs/VG/CC	Aptamer-PolyTCuNPs	10–2200 nM	3.5 pM	Serum	[116]
	Aptamer/AuNPs/GCE	CuMOFs/AuNPs-Aptamer	1 nM–2 μM	0.45 nM	A-CSF	[117]
	Aptamer/AuE	AgNPs	1 pM–10 nM	430 fM	Serum	[118]
	Aptamer/Au-MXene	Aptamer-TB-Au@COFs	0.01–180 pg/mL	4.27 fg/mL	Serum	[119]
	Aptamer/COF/GCE	HRP-PrP^C^	0.01–2.5 nM	2.17 fM	CSF	[120]
Peptide	PrP^C^/AuNPs/GCE	CH-Cu-NAs-Aptamer	0.05–5 nM	19.2 pM	Serum	[121]
	PrP^C^/ITO	CdTe QDs-Aptamer	0.5 pM–1000 nM	0.5 pM	Serum	[122]
	PrP^C^/Au@pCOFs/GCE	MB@aCOFs-Aptamer	10 pM–10 μM	5.1 pM	CSF	[123]
	GCE	Th@AuNPs-Aptamer	0.5–30 nM	100 pM	A-CSF	[124]

Abbreviations: GCE, glass carbon electrode; C_3_N_4_, carbon nitride; CoS_2_DSNBs, CoS_2_ double shelled nanoboxes; Ag NPs, silver nanoparticles; CNT, carbon nanotube; PIL, polymeric ionic liquid; NLC, nanostructured lipid carrier; Th, thionine; SiC, silicon carbide; PEDOT NWs, poly(3,4-ethylene dioxythiophene) nanowires; BPQDs, black phosphorus quantum dots; ITO, indium tin oxide; AuNRs, gold nanorods; AuNPs/VG/CC, Au nanoparticles-modified vertical graphene/carbon cloth; PolyTCuNPs, poly T-templated copper nanoparticles; CuMOFs, copper-based metal–organic frameworks; TB, toluidine blue; COF, covalent organic framework; HRP, horseradish peroxidase; PrP^C^, prion protein; CH-Cu-NAs, laccase-mimicking nanozyme assemblies; MB, methylene blue; A-CSF, artificial cerebrospinal fluid.

## 4. Conclusions

In summary, we have reviewed the progress in (photo)electrochemical biosensors used for AβO detection and discussed the analytical principles and recognition elements. Compared with traditional methods and technologies, (photo)electrochemical biosensors in direct and sandwich-like detection ways have the characteristics of high sensitivity, fast response, and simple operation. Direct methods are simpler, faster, and lower in cost, but generally offer moderate sensitivity. Sandwich assays employing a pair of recognition elements and various signal reporters (nanomaterials, enzymes, DNA amplifiers) achieve substantially lower detection limits. Among transduction techniques, ECL and PEC are the most sensitive, outperforming conventional EC methods by 2–3 orders of magnitude. Therefore, for ultrasensitive AβO detection, sandwich-type ECL/PEC biosensors using aptamers or antibodies are recommended. For cost-effective and rapid screening, direct EC methods with peptides or aptamers are more suitable. The used recognition elements for the capture and identification of AβO include antibodies, aptamers, peptides, and MIPs. With the advancement of materials science, the development of novel synthetic receptors with high affinity, stability, and specificity has become possible, such as dual-recognition units by integrating aptamers and MIPs. Multi-component nanomaterials with different sizes, shapes, chemical compositions, and surface modifications have been used to fabricate high-performance biosensors. Rational selection for AβO biosensing should be guided by several key criteria: (1) high specific surface area and well-defined porous architecture are essential for high-density immobilization of recognition elements and efficient loading of signal reporters; (2) materials must be stable under the measurement conditions, such as aqueous buffer with neutral pH, appropriate redox potential, and existence of additional redox species; (3) low cytotoxicity is critical for future clinical translation, especially when the sensor is intended for point-of-care or implantable applications. Carbon-based materials and biocompatible MOFs are generally less toxic than heavy metal-containing nanomaterials; (4) high conductivity can facilitate electron transfer and enhance signal transduction. MXenes and some conductive COFs or MOFs with metal catenation or conjugated backbones are advantageous for electrochemical sensing. Poorly conductive materials require co-modification with conductive components like AuNPs or carbon nanotubes.

Development and clinical translation of nanomaterial-based AβO biosensors face some challenges. Slight differences in nanoparticle size, shape, or surface functionalization during synthesis may significantly alter electron transfer kinetics and probe immobilization efficiency, leading to poor reproducibility across biosensor batches. Many nanomaterials are prone to oxidation, aggregation, or degradation in aqueous buffers or biological fluids, reducing shelf life and causing signal drift. Residual reagents and heavy metal ions or reactive oxygen species generated by some nanomaterials may induce cytotoxicity, limiting their application in point-of-care or implantable devices. In addition, AβO is highly unstable and prone to form higher-order assemblies, such as proto-fibrils, fibrils, and plaques. It is urgent to develop biosensors for fast and high-throughput analysis of AβO and other AD-related biomarkers to improve diagnostic accuracy. The customization and miniaturization of electrochemical instruments and their integration with microfluidic technology are the development trends for faster and better multiplex detection in clinical diagnosis. In addition, most of the AβO species exhibit similar antiparallel β-sheet structures, making it difficult to effectively differentiate these oligomeric intermediates with traditional recognition elements, such as antibodies and peptides. It is necessary to design and produce more novel and effective recognition elements to identify and determine the level of multiple AβO species. Furthermore, translational barriers hinder the clinical translation of (photo)electrochemical AβO biosensors. Matrix effects in real cerebrospinal fluid or blood, such as high-abundance proteins and lipids, can interfere with specific recognition, block electrode surfaces, and disrupt the stability of AβO. Biofouling and non-specific adsorption may increase background impedance and cause false signals, especially in label-free EIS measurement. Although antifouling coatings such as zwitterionic peptides, polyethylene glycol, and hydrogel have shown promise, their validation in undiluted clinical samples remains limited. In addition, there is a lack of universally recognized gold standards for the in vitro preparation of AβO samples and the construction of biosensors, such as incubation conditions, electrode modification procedures, recognition elements, and testing parameters. Significant differences in experimental parameters among different laboratories will result in significant batch heterogeneity in the detection results. Artificial intelligence can significantly improve the real-time predictive accuracy of biosensors by rapidly analyzing large datasets, efficiently detecting intricate patterns, subtle trends, and irregularities. This technique will help researchers make immediate decisions under dynamically changing conditions. Through the combination with artificial intelligence, we believe that biosensors will provide a bright prospect for the diagnosis and treatment of AD.

## Data Availability

Not applicable.
